# Evaluating the World Health Organization’s SkinNTDs App as a Training Tool for Skin Neglected Tropical Diseases in Ghana and Kenya: Cross-Sectional Study

**DOI:** 10.2196/51628

**Published:** 2024-04-30

**Authors:** Mireia Cano, José A Ruiz-Postigo, Paul Macharia, Yaw Ampem Amoako, Richard Odame Phillips, Esther Kinyeru, Carme Carrion

**Affiliations:** 1 eHealth Lab Research Group eHealth Center, School of Health Sciences Universitat de Catalunya Barcelona Spain; 2 Innovation, Digital Transformation and Health Economics Research Group Research Institut Germans Trias i Pujol Badalona Spain; 3 Prevention, Treatment and Care Unit Department of Control of Neglected Tropical Diseases World Health Organization Geneva Switzerland; 4 Strathmore University Nairobi Kenya; 5 Kumasi Centre for Collaborative Research in Tropical Medicine Kwame Nkrumah University of Science and Technology Kumasi Ghana; 6 School of Medicine and Dentistry Kwame Nkrumah University of Science and Technology Kumasi Ghana; 7 Ministry of Health Nakuru Kenya; 8 School of Health Sciences Universitat de Girona Girona Spain; 9 Network for Research on Chronicity, Primary Care, and Health Promotion Barcelona Spain

**Keywords:** SkinNTDs app, mHealth, mobile health, neglected tropical diseases, NTDs, skin neglected tropical diseases, low- and middle-income countries, tropical disease, app, training tool, digital health, intervention, decision support tool, mobile phone

## Abstract

**Background:**

Neglected tropical diseases (NTDs) affect over 1.5 billion people worldwide, primarily impoverished populations in low- and middle-income countries. Skin NTDs, a significant subgroup, manifest primarily as skin lesions and require extensive diagnosis and treatment resources, including trained personnel and financial backing. The World Health Organization has introduced the SkinNTDs app, a mobile health tool designed to train and be used as a decision support tool for frontline health care workers. As most digital health guidelines prioritize the thorough evaluation of mobile health interventions, it is essential to conduct a rigorous and validated assessment of this app.

**Objective:**

This study aims to assess the usability and user experience of World Health Organization SkinNTDs app (version 3) as a capacity-building tool and decision-support tool for frontline health care workers.

**Methods:**

A cross-sectional study was conducted in Ghana and Kenya. Frontline health care workers dealing with skin NTDs were recruited through snowball sampling. They used the SkinNTDs app for at least 5 days before completing a web-based survey containing demographic variables and the user version of the Mobile Application Rating Scale (uMARS), a validated scale for assessing health apps. A smaller group of participants took part in semistructured interviews and one focus group. Quantitative data were analyzed using SPSS with a 95% CI and *P*≤.05 for statistical significance and qualitative data using ATLAS.ti to identify attributes, cluster themes, and code various dimensions that were explored.

**Results:**

Overall, 60 participants participated in the quantitative phase and 17 in the qualitative phase. The SkinNTDs app scored highly on the uMARS questionnaire, with an app quality mean score of 4.02 (SD 0.47) of 5, a subjective quality score of 3.82 (SD 0.61) of 5, and a perceived impact of 4.47 (SD 0.56) of 5. There was no significant association between the app quality mean score and any of the categorical variables examined, according to Pearson correlation analysis; app quality mean score vs age (*P*=.37), sex (*P*=.70), type of health worker (*P*=.35), country (*P*=.94), work context (*P*=.17), frequency of dealing with skin NTDs (*P*=.09), and dermatology experience (*P*=.63). Qualitative results echoed the quantitative outcomes, highlighting the ease of use, the offline functionality, and the potential utility for frontline health care workers in remote and resource-constrained settings. Areas for improvement were identified, such as enhancing the signs and symptoms section.

**Conclusions:**

The SkinNTDs app demonstrates notable usability and user-friendliness. The results indicate that the app could play a crucial role in improving capacity building of frontline health care workers dealing with skin NTDs. It could be improved in the future by including new features such as epidemiological context and direct contact with experts. The possibility of using the app as a diagnostic tool should be considered.

**International Registered Report Identifier (IRRID):**

RR2-10.2196/39393

## Introduction

### Background

The World Health Organization (WHO) defines mobile health (mHealth) as the application of wireless technology, such as mobile phones, to the provision of health care. It is regarded as a component of eHealth, which also refers to the secure and cost-effective use of information and communication technology to assist with health system and disciplines related with health [1]. In fact, the WHO itself has recognized that eHealth has the potential to significantly contribute to sustainability and accessibility in the health system. In this regard, the number of mHealth interventions worldwide has been steadily increasing over the last decade.

This is even more relevant in regions such as Africa where the health challenges are much greater. In 2022, a systematic review of digital health interventions in sub-Saharan Africa identified 738 digital health interventions, highlighting multiple overlapping solutions with limited focus and scalability [2]. The WHO is therefore working to address these challenges, and already in 2010, it published a continent-specific digital health strategy [3].

Among the diseases that seem likely to benefit from such interventions are the neglected tropical diseases (NTDs) [4], a group of 20 diseases and conditions identified by the WHO. As their name implies, NTDs are diseases that have not historically received any kind of priority attention from international health organizations, despite affecting more than 1.5 billion people [5]. This is directly related to the fact that most of these illnesses are only found in low- and middle-income countries (LMICs) in tropical and subtropical regions, and they primarily affect women and children [5].

If undetected or untreated, some of these diseases can either be fatal or become chronic and irreversible, leading not only to lifelong disabilities but also compromised mental well-being; stigmatization; social exclusion; and, in certain countries, even experiences of racism [6-8]. This in turn perpetuates a cycle of poverty that has a direct impact on the development and economic productivity of LMICs [6].

In 2005, the WHO finally decided that a comprehensive strategy was needed to address the complexity surrounding these diseases. It therefore shifted from tackling them individually to tackling them as a single group, the NTDs [5]. It later decided to create subgroups depending on their management: those that are potentially preventable through large-scale chemotherapy interventions and those that require individual case management [9]. Within the latter category lie skin NTDs, a group of NTDs that manifest primarily as skin lesions, such as edema, patches, and ulceration, which can be detected through visual screening [10]. This group consists of Buruli ulcer, cutaneous leishmaniasis, deep fungal infections, post–kala-azar dermal leishmaniasis, leprosy, lymphatic filariasis, mycetoma, onchocerciasis, scabies and other ectoparasites, and yaws. The management of these diseases hinges on early detection and treatment, which demands significant resources, including skilled personnel and financial support [11,12]. However, early detection of these diseases is often difficult due to various factors. For instance, they are frequently painless, which can prevent them from seeking medical attention at an early stage. In addition, there is low awareness of these diseases among the population at highest risk, and, as mentioned before, stigma and discrimination associated with these diseases can also discourage people from seeking medical help [11,12].

Therefore, a key player that emerges in the strategic framework for successfully managing skin NTDs are the *frontline health workers*. This term refers to any health worker who directly provides service to a community. Although they frequently lack specialized medical training, knowledge of data collection techniques, and peer contact, they are real key players [11,12]. Indeed, they often are the initial point of contact for disease control for most skin NTDs, given that they can be visually identified and that clinical diagnosis is the most accessible diagnostic tool available [11]. Hence, it is important to enhance their education and training to facilitate their role in diagnosing, in treating, in referring patients to another level of the health system [10], and even in mitigating the stigma associated with skin NTDs [13]. In order to achieve this last-mentioned goal, numerous training initiatives for frontline health workers have been undertaken to date with good results and have been well received by them [11,13-17]. However, the predominance of in-person small group formats, with the program often targeting only 1 condition, limits the reach and increases the expense of these initiatives. Identifying alternatives to enhance the efficiency and expandability of these programs is crucial [11]. mHealth stands out as a promising, practical, and extendable approach to support frontline health worker training [18]. Although its adoption for this specific purpose has been limited, reviews of existing literature have highlighted its potential effectiveness [18]. Furthermore, specifically for skin NTDs, the WHO promotes the use of teledermatology and web-based training courses and materials whenever possible. That is the main reason why the WHO’s Department of Control of Neglected Tropical Diseases has developed the SkinNTDs app [19], a mobile version of the training guide they published in 2018 [20]. By using an algorithm based on identifying signs and symptoms and providing more information about these diseases, this app assists frontline health workers in the diagnosis and management of skin NTDs.

However, although the third version has already been released, the app’s usability, efficacy, and effectiveness have not yet been evaluated. This is a critical step in guaranteeing quality and empowering end users not to rely entirely on popularity or “star”-rating systems, which have already been proved insufficient [21]. Evaluation of mHealth interventions is a key component of most digital health technology frameworks [22-24], even more so when app-based mHealth interventions are scarce in Africa, as most mHealth interventions in this region are SMS text messaging based [25].

The intervention maturity life cycle schematic proposed by WHO [24] is a practical guide explaining the goals at each stage, the number of participants required, and the measurement targets. Considering these guidelines and the stage of maturity of the SkinNTDs app (the first and second stage), it now seems appropriate to evaluate its feasibility and usability. In this stage, questions regarding how the app is used by end users, how it fits into their workflow, and how easy the learning curve of use is should be answered before moving on to the next stage (efficacy).

One reliable tool available to assess usability is the user version of the Mobile Application Rating Scale (uMARS) [26], a simple tool for classifying and rating mHealth apps based on 4 objective subdomains (engagement, functionality, aesthetics, and information quality) and 1 subjective quality subdomain.

### Objective

Given the WHO’s commitment to mHealth as a tool to achieve a range of sustainable goals, it is essential to evaluate interventions that have the potential to be highly scalable and cost-effective. This paper summarizes the results of a cross-sectional study assessing the engagement, functionality, aesthetics, and information quality of version 3 of the SkinNTDs app for the real end user in their actual context according to a validated tool. In addition, a secondary objective was to check whether the demographic information gathered influenced the final uMARS score, given that the developers of the SkinNTD app had no plans to customize the app for different settings upon its implementation.

## Methods

### Study Design

A cross-sectional study was conducted between December 2022 and April 2023 in Ghana and Kenya. The study design and methods are described in detail in the published protocol [27]. Deviations from this protocol are summarized in Multimedia Appendix 1.

### Ethical Considerations

This study was conducted according to the ethical principles established by the World Medical Association in the Declaration of Helsinki of Ethical Principles for Medical Research Involving Human Subjects [28]. To guarantee that the protocol complied with the ethical standards of all 3 countries involved, it was approved by their respective ethics committees. The protocol was authorized by the Ethics Committee of Universitat Oberta de Catalunya (Spain) (20201127_mcarrion_NTDs), the Ethical Committee of Kwame Nkrumah University of Science and Technology (Ghana) (CHRPE/AP/576/22), and the Ethical Committee of Coast General Teaching and Referral Hospital (Kenya) (ERC-CGH/MSc/VOL.I).

Participants gave informed consent to participate in the study before taking part. To ensure confidentiality and anonymity, each participant was assigned a unique identification number (eg, P01) for coding all collected information and data. Moreover, participants did not receive any economic compensation for their participation in this study.

### Participant Recruitment and Eligibility Criteria

Nonprobabilistic snowball sampling was used due to the difficulty in making direct contact with frontline health care workers in these 2 countries. Snowball sampling is a recruitment technique in which chosen participants are asked to find and contact other potential participants from among their acquaintances. Although this approach is nonrandomized, it appeared to be the best strategy to locate the participants of this study, as the main researchers were based on a different continent.

**Figure 1:**
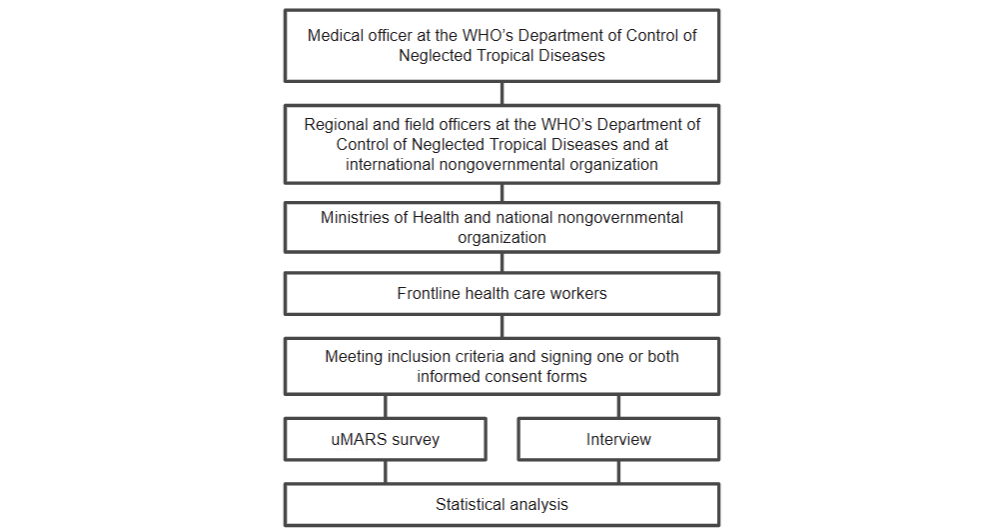
Diagrammatic representation of snowball sampling recruitment. MARS: Mobile Application Rating Scale; WHO: World Health Organization.

A diagram representation of this snowball sampling recruitment is shown in Figure 1. More detailed information can be found in the published protocol [27].

Anyone who worked or had worked with skin NTDs on a regular basis in the 2 selected countries, who were or had been in charge of their diagnosis and treatment, who had a smartphone (Apple or Android), and who had downloaded and used the SkinNTDs app on at least 5 different days was eligible to take part. In addition, participants had to use WhatsApp (Meta Platforms) or email to send the informed consent. Limited understanding of the English language and refusal to sign informed consent form were factors for exclusion.

The same population was the target for both parts of the study. Before signing the informed consent form, participants were asked to read the information page.

### Sample Size Calculation

As already mentioned, this study followed the steps proposed in the intervention maturity life cycle schematic described by the WHO in *Monitoring and Evaluating Digital Health Interventions* [24]. As a result, sample size was determined using this guide.

The SkinNTDs app is now at the prototype stage, which, in accordance with WHO recommendations, corresponds to stages 1 and 2. The WHO suggests evaluating the intervention with a sample size between 10 and 100 people. Given that a sample of 100 participants is the maximum number advised and that this study was conducted on the web, we assumed a 50% dropout rate. These calculations resulted in a 50-person final sample size.

### Outcomes

#### Demographic Variables

Participants were asked to complete an anonymous survey of various demographic data that included age, sex, country of residence, type of frontline health care worker, frequency of dealing with skin NTDs, experience and training in dermatology, work environment, working institution, knowledge of mobile technology, and languages spoken.

#### Quantitative Method: uMARS Questionnaire

The uMARS was used to assess the quality of the SkinNTDs app [26]. This tool assesses 20 items clustered into 4 objective subscales (engagement, functionality, aesthetics, and information quality) and 1 subjective subscale. Participants rated each item using a Likert scale from 1 to 5; higher numbers meant a better rating. A “not applicable” option was available in case an item could not be assessed. In the extra “app-specific” category, only the relevant questions for this study were chosen. Finally, 9 self-created questions were added to complete the survey.

A total of 2 scores were obtained based on the original recommendation from the authors of this scale: app quality score and app subjective quality score. Questions rated as not applicable were excluded from the score.

In our study, we adapted the evaluation process based on the original article’s recommendations, which suggested a minimum application use time of 10 minutes before completing the uMARS. However, we considered that this time was not enough to complete a comprehensive evaluation, and we extended to 5 days.

#### Qualitative Methods: Semistructured Interviews and Focus Groups

Due to the different realities of the context of the 2 countries, 2 qualitative methods were used.

Semistructured interviews were conducted as a qualitative method for exploring the perspectives, perceptions, and opinions of participants, combining prepared questions with others that arose during the interview [29]. Each participant was asked 7 questions based on the key findings obtained from the uMARS questionnaire in the same order and in the same words as a standardization that facilitated comparison [30]. In addition, interviewers could ask unforeseen questions based on a participant’s answer. The questions were unbiased, open-ended, and well-written and used simple terms.

Focus groups were also used as another qualitative research method for the same purpose as before. This method involves a moderator posing targeted questions to participants in a group setting, with the advantage of capturing valuable interactions among them [31]. Focus group sessions also incorporated the same set of 7 questions used in the semistructured interviews. Moreover, the moderator had the same flexibility to ask unforeseen questions based on the participants’ interactions and responses.

Our study design incorporated a predetermined objective to include a minimum of 10% of the overall sample size or continue data collection until reaching a point of information saturation using both methods.

More details of the instruments can be found in the study protocol [27].

### Data Collection and Study Procedure

The principal researcher delivered the consent form via email or WhatsApp when frontline health care workers contacted her. However, participants enrolled at Coast General Teaching and Referral Hospital (Kenya), due to ethical requirements, signed a paper-based consent form delivered to them by the local investigator. After it was signed, participants received a follow-up email with more details about the study and a link to the uMARS survey, which they had to answer after using the app for at least 5 days.

Participants were able to express their willingness to take part in the semistructured interviews in the same questionnaire.

After reading the detailed information sheet for this second phase of the study, participants who had agreed were required to sign a second consent form. Then, the interview was scheduled by mutual agreement.

The interviews were conducted via Google Meet (Google) and had a duration ranging from 25 to 40 minutes. The focus groups were conducted face-to-face and moderated by a local researcher for 60 minutes. All sessions were recorded regardless of the qualitative method used.

During this time, the interviewer and moderator could take notes and reiterate any questions or sentences for clarification.

Interviews and focus groups were all transcribed using Otter.ai software (Sam Liang) for analysis. The research protocol provides more information on the procedure for data collection [27].

### Statistical Analysis

All quantitative data analyses were conducted using SPSS (version 25; IBM Corp) for Windows, with the statistical significance set at *P*≤.05. A descriptive analysis was conducted to describe the demographic variables and data obtained from the uMARS scores. Categorical variables were described in terms of frequencies and continuous variables in terms of central tendency and dispersion measurements. All data were shown in tables. Data normality was evaluated using Kolmogorov-Smirnov test (*P*=.05). The *t* test (2-tailed) was used for comparing means of bivariate variables and ANOVA for comparing means of multivariate variables. A logistic regression analysis was performed to add the covariates that could skew the main association under analysis. A CI of 95% was assumed, and *P*≤.05 was considered a significant difference.

Selected quotes were returned to participants for approval. Qualitative data derived from the semistructured interviews and focus groups were analyzed using ATLAS.ti (ATLAS.ti Scientific Software Development GmbH). We identified attributes, clustered them into different themes, and then coded these themes to analyze the various dimensions explored during the interviews.

## Results

### Participants

In total, 60 participants took part in this study. Demographic characteristics are shown in Table 1.

Of the 60 participants, 57% (n=34) were male individuals. Participants were divided quite homogeneously in the different age groups, <36 years (n=22, 37%), 36 to 45 years (n=22, 37%), and 46 to 65 years (n=16, 27%). Participation was higher in Kenya with 36 (60%) participants, compared with the 24 (40%) from Ghana. Up to 93% (n=56) of them referred to working in a public health care institution. From all these participants, 72% (n=43) identified themselves as frontline health care workers, and 62% (n=37) mentioned working in a rural area. The vast majority of the participants were not dermatology specialists but said they had some experience with the topic (n=44, 73%). The participants were distributed similarly in terms of how frequently they dealt with skin. More than half of the participants (n=41, 68%) considered they had extensive knowledge of mobile technology, and 87% (n=52) had no additional training before using the app. Speaking English was a requirement for participation, so everyone who participated did. However, there were up to 19 more languages registered, among which Swahili stood out as the second most spoken language by the participants in Kenya (25/36, 69%) and Twi in Ghana (20/24, 83%).

**Table 1:** Participant demographics in the World Health Organization’s SkinNTDs app usability and user experience assessment through the user version of the Mobile Application Rating Scale survey (n=60) shown by frequencies (%).

Variable				Participants, n (%)
**Age (y)**				
	<35			22 (37)
	36-45			22 (37)
	46-65			16 (27)
**Sex**				
	Female			26 (43)
	Male			34 (57)
**Country**				
	Kenya			36 (60)
	Ghana			24 (40)
**Type of health worker**				
	Frontline			43 (72)
	Nonfrontline			17 (28)
**Work context**				
	Rural			37 (62)
	Urban			23 (38)
**Type of working institution**				
	Public health care setting			56 (93)
	Private health care setting			2 (3)
	Nongovernmental organization			2 (3)
**Dermatology experience**				
	Not trained, no experience in dermatology			13 (22)
	Not trained, but some experience in dermatology			44 (73)
	Trained and experienced in dermatology			3 (5)
**Frequency of dealing with skin** **neglected tropical diseases** **(cases/month)**				
	Rarely (<1)			13 (22)
	Occasionally (1-3)			13 (22)
	Frequently (4-6)			21 (35)
	Usually (>6)			13 (22)
**Knowledge of mobile technology**				
	High knowledge			41 (68)
	Medium knowledge			19 (32)
**Extra training in app use**				
	Yes			8 (13)
	No			52 (87)
**Language**				
	**Kenyan participants (n=36)**			
		English	36 (100)	
		Swahili	25 (69)	
		Kamba	3 (8)	
		Kikuyu	2 (6)	
		Marakwet	2 (6)	
		Dhulo	1 (3)	
		Kisii	1 (3)	
		Kalenjin	1 (3)	
		Oromo	1 (3)	
		Somali	1 (3)	
	**Ghanaian participants (n=24)**			
		English	24 (100)	
		Twi	20 (83)	
		Asanti	3 (12)	
		Fante	2 (8)	
		Akan	1 (4)	
		French	1 (4)	
		Chinese	1 (4)	
		Ga	1 (4)	
		Ewe	1 (4)	

### uMARS Score

Tables 2 and 3 show the overall results from the 3 sections of the uMARS questionnaire and specific results for each subdomain. They appear separately according to the recommendations of the uMARS authors, in order to strengthen the objectivity of the final result.

The SkinNTDs app received an overall score of 4.02 (0.47) out of 5 in the app quality mean score, 3.82 (0.61) out of 5 in the subjective mean score, and 4.47 (0.56) out of 5 in the perceived impact section. Thus, the app received more than 75% of the maximum score in all 3 sections.

These overall scores can be broken down as follows: starting with the most objective scoring, the app quality mean score is the average obtained in each of the 4 uMARS domains (engagement, functionality, aesthetics, and information quality), which in turn are divided into 16 subdomains. The scores range from 3.65 to 4.20, with engagement being the lowest rated and information quality the highest rated. Moreover, SDs are between 0.47 and 0.65, indicating that the dispersion of the data tends to be medium-low. Notably, the lowest-rated subdomain was customization (mean 3.18, SD 1.02, out of 5), which refers to whether the app provides all necessary settings and preferences for app features (eg, sound, content, and notifications). In contrast, the credibility of the source subdomain was the highest rated of the 16 (mean 4.55 (SD) out of 5), referring to whether the app comes from a legitimate source.

At a subjective level, the app obtained a mean score of 3.82 (SD) out of 5. The findings in this section indicate that individuals are not very eager to pay for this app (mean 2.57, SD 1.33); however, the subvariable measuring whether they would recommend the app had a very high score (mean 4.65, SD 0.70). Finally, when the participants were asked for the overall rating, the average score was 4.08 (SD), which is extremely close to the quality mean score of 4.02 (SD). In this case, the SDs are slightly higher than in the previous section, indicating more variability in the responses.

Finally, the participants rated the perceived impact score (including awareness and knowledge) highly, with mean values of 4.40 (SD) and 4.55 (SD) out of 5 correspondingly.

Table 3 shows more detailed information regarding the specific results for each subdomain. In addition, graph 1 presents the scores of all objective domains assessed in a visual way.

**Figure 2:**
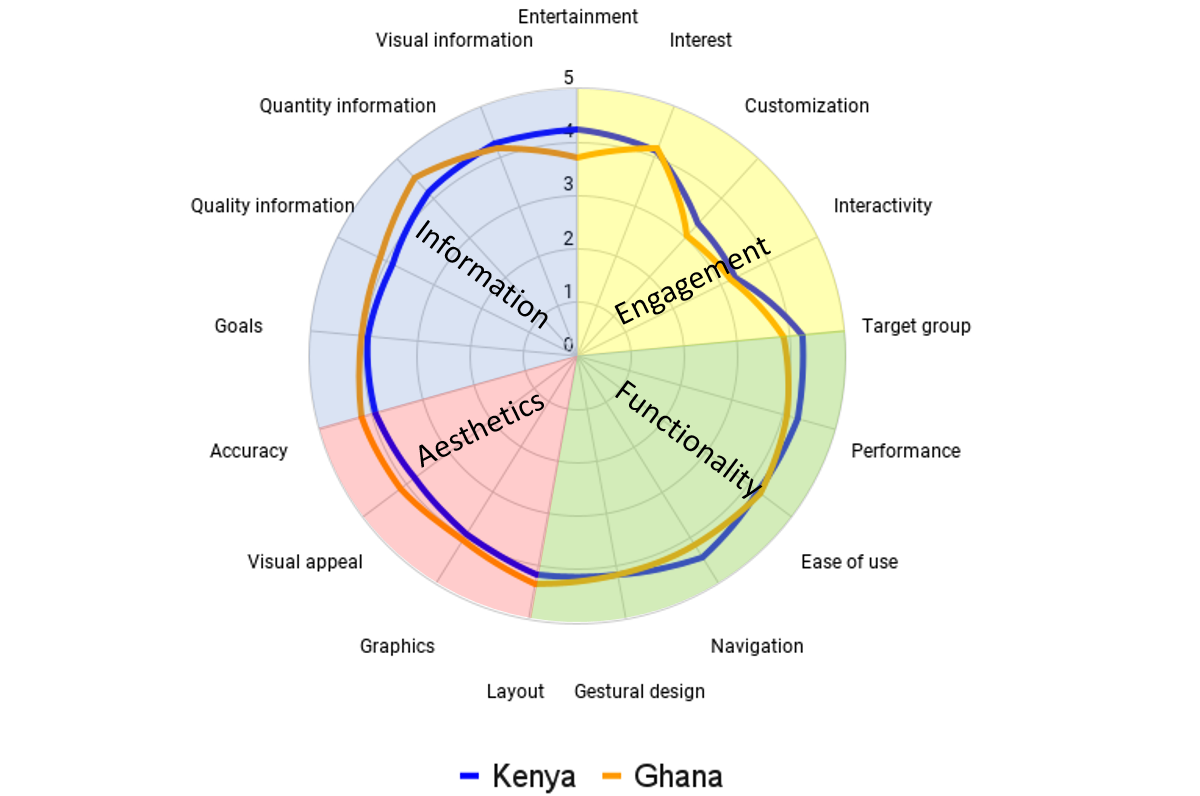
Radar chart evaluation of each app’s objective quality domain according to the user version of the Mobile Application Rating Scale (uMARS), with results divided by country.

Table 3 specifies the detailed results for each subdomain. In addition, Figure 2 shows the results obtained in each subdomain visually by country.

The study was completed with the comparison of the mean, which was based on the comparison between the global app quality mean score versus the main domains. The Kolmogorov-Smirnov test demonstrates a normal distribution of the data derived from the app quality mean score. Thus, a *t* test was used for comparing means of bivariate variables and ANOVA for comparing means of multivariate variables.

**Table 2:** Results of the validated user version of the Mobile Application Rating Scale domains according to the cross-sectional study.

Domain		Mean (SD)	Minimum	Maximum
**App quality mean score**		4.02 (0.47)	2.89	4.83
	Engagement mean score	3.65 (0.57)	2.20	4.80
	Functionality mean score	4.18 (0.65)	2.50	5
	Aesthetics mean score	4.02 (0.53)	3	5
	Information mean score	4.20 (0.49)	3	5
App subjective mean score		3.82 (0.61)	2.25	5
App perceived impact		4.47 (0.56)	2.50	5

**Table 3:** Results of each validated user version of the Mobile Application Rating Scale subscale domain according to the cross-sectional study.

Subdomains			Mean (SD)		Minimum		Maximum
**Engagement score**							
	Entertainment	4.03 (0.69)		3		5	
	Interest	4.08 (0.72)		2		5	
	Customization	3.18 (1.02)		1		5	
	Interactivity	3.22 (0.92)		1		5	
	Target group	3.98 (0.97)		1		5	
**Functionality score**							
	Performance	4.17 (0.93)		2		5	
	Ease of use	4.20 (0.71)		3		5	
	Navigation	4.30 (0.83)		2		5	
	Gestural design	4.15 (0.71)		2		5	
**Aesthetics score**							
	Layout	4.20 (0.73)		3		5	
	Graphics	3.93 (0.68)		3		5	
	Visual appeal	3.90 (0.54)		3		5	
**Information score**							
	Quality of information	3.93 (0.63)		2		5	
	Quantity of information	4.32 (0.75)		2		5	
	Visual information	4.18 (0.65)		3		5	
	Credibility of source	4.55 (0.80)		2		5	
**Subjective items score**							
	Would you recommend?	4.65 (0.75)		2		5	
	How many times?	3.98 (0.83)		2		5	
	Would you pay?	2.57 (1.33)		1		5	
	Overall (star) rating	4.08 (0.70)		2		5	
**Perceived impact score**							
	Awareness	4.40 (0.59)		3		5	
	Knowledge	4.55 (0.62)		2		5	

The results (Table 4) revealed that there was no statistically significant difference in any of the variables.

Similarly, linear regression showed that there was no significant association between the app quality mean score and any of the categorical variables examined above (*F*=0.60; *P*=.76), with *R*^2^=0.07.

The linear regression model analyzing the main demographic variables of the participants and their correlation with the user version of the Mobile Application Rating Scale (uMARS) app quality mean score in the cross-sectional study is as follows: The model shows an R value of 0.27, an R^2^ of 0.07, and an adjusted R^2^ of –0.05. The *F* test for this model (*df*=0.60) resulted in a *P* value of .76. The dependent variable for this analysis was the app quality mean score, and the predictors included age, sex, type of health worker, country, work context, frequency of dealing with skin NTDs, and dermatology experience. Meaning that there was no significant association between the app quality mean score and any of the categorical variables examined. Finally, as mentioned in the Methods section, 7 questions were added to complete the app evaluation, which are shown in Table 5.

It is worth noting that 64% (36/60) of participants used the app more than the minimum number of times required before answering the survey, and the majority believed that using the app would reduce the time it takes to diagnose skin NTDs. In terms of connectivity, while up to 67% (40/60) claimed to have a strong connection, a substantial minority group of 33% (20/60) still had limited or no access to the internet.

Almost all the participants favored the inclusion of skin NTDs surveillance, the ability to include patient records, and the availability of a computer version of the app.

Slightly more than half of the participants (32/60, 53%) considered it necessary for the app to be available in other languages, with Twi and Swahili being the first on the list.

**Table 4:** Comparison of means between user version of the Mobile Application Rating Scale app quality mean score versus the main demographic variables of participants in the cross-sectional study.

Variables compared and statistical tests performed		Value	*P* value
**App quality mean score vs age**			
	Levene statistic	1.42	.25
	ANOVA	1.01	.37
**App quality mean score vs sex**			
	Levene statistic	2.24	.14
	*t* test (*58*)	0.40	.70
**App quality mean score vs type of health worker**			
	Levene statistic	0.04	.84
	*t* test (*58*)	–0.94	.35
**App quality mean score vs country**			
	Levene statistic	5.09	.03
	T-Welch	0.08	.94
**App quality mean score vs work context**			
	Levene statistic	2.95	.09
	*t* test (*df*)	–1.40 (58)	.17
**App quality mean score vs frequency of dealing with skin neglected tropical diseases**			
	Levene statistic	4.13	.01
	Kruskal-Wallis test	6.40	.09
**App quality mean score vs dermatology experience**			
	Levene statistic	0.88	.50
	ANOVA	0.70	.63

**Table 5:** Other relevant questions added to the user version of the Mobile Application Rating Scale survey (n=60).

Questions			Responses, n (%)
**Duration of use**			
	<7 days	21 (35)	
	1-4 weeks	26 (43)	
	1-2 months	11 (18)	
	2-4 months	2 (3)	
**Internet connection**			
	I hardly have internet access	3 (5)	
	Most of the time I do not have internet access	8 (13)	
	Sometimes I have internet access	9 (15)	
	Most of the time I have internet access	31 (52)	
	I always have internet access	9 (15)	
**Translation**			
	Yes	32 (53)	
	No	28 (47)	
**Surveillance**			
	Yes	100 (100)	
**Patient records**			
	Yes	59 (98)	
	No	1 (2)	
**Desktop version**			
	Yes	51 (85)	
	No	9 (15)	

### Semistructured Interviews

#### Overview

Regarding the second part of the study, 55% (33/60) of participants showed an interest in participating. However, only 13 men and 4 women were interviewed (5 from Ghana and 12 from Kenya). Upon interviewing 17 people, we observed that the responses started to repeat, indicating data saturation. The data collection comprised 10 semistructured interviews, with 5 participants from Ghana and 4 from Kenya, along with a single focus group conducted in Kenya involving 8 participants.

Most participants (10/17, 59%) were aged between 46 and 65 years. Approximately, 76% (13/17) of the participants were frontline health care workers, and 47% (8/17) said that they had occasional contact with skin NTDs. Demographic characteristics are shown in Table 6.

Despite being asked 7 questions, not all participants were able to provide detailed answers that allowed attributes to be identified. This was mainly due to technological and language barriers.

Both the semistructured interview and the focus group data were transcribed by 2 researchers (MC and PM) and thoroughly reviewed by 1 researcher (MC) to gain an initial understanding of the data. Subsequently, expressions aligning with the study objectives were extracted from the text and transformed into attributes (n=95) using ATLAS.ti software. These codes represented condensed versions of the professionals’ thoughts. On the basis of the content similarity, the codes were further grouped into themes (n=18), which were then associated with each question asked. At this stage, another researcher (CC) examined the coding, subcategories, and higher-level categories. Discussions were held to reach a consensus on the content and names assigned to categories.

Table 7 presents the description and frequency of the identified themes and selected participant quotes.

**Table 6:** Participant demographics in the World Health Organization’s SkinNTDs app usability and user experience assessment through semistructured interviews and focus group (n=17).

Variable			Participants, n (%)
**Age (y)**			
	<35	4 (23.5)	
	36-45	3 (17.6)	
	46-65	10 (58.8)	
**Sex**			
	Female	4 (23.5)	
	Male	13 (76.5)	
**Country**			
	Kenya	12 (70.6)	
	Ghana	5 (29.4)	
**Type of health worker**			
	Frontline	13 (76.5)	
	Nonfrontline	4 (23.5)	
**Frequency of dealing with skin neglected tropical diseases (cases/month)**			
	Rarely (<1)	4 (23.5)	
	Occasionally (1-3)	8 (47.1)	
	Frequently (4-6)	3 (17.6)	
	Usually (>6)	2 (11.8)	
**App assessment duration (5 days)**			
	Yes	14 (82.4)	
	No	3 (17.6)	

**Table 7:** Description and frequency of the identified themes and selected participant quotes.

Theme		Participants^a^, n (%)	Best points
**Best points of the SkinNTDs app (n=10)**			
	Good for people without SkinNTDs experience	8 (80)	“The App gives you a wide understanding about SkinNTDs without being an expert in this topic.” (P1) “The App helps people without knowledge in SkinNTDs and leads them to the right directions to make diagnosis.” (P3)
	Ease of use	3 (30)	“What I really appreciate from the App is that is really easy to navigate.” (P3)
	Friendly interface	4 (40)	“I really like the interface.” (P4)
	Offline functionality	4 (40)	“Is nice to have this App always available with you, even when you don’t have access to internet.” (P2)
	Easy accessibility	4 (40)	“The fact that this App works offline, it means it is portable. And even in the most remote places it is still accessible. Making in a huge plus for its spread use.” (P4) “It is nice that the App can be accessed anytime.” (P9) “The app is always accessible.” (P8)
	Simple language	2 (20)	“It is easy to understand because uses simple English.”(P9)
	Scope	2 (20)	“So it is good because, many people can be able to get the app and use it.” (P14) “I think the app can be used especially in those areas, remote areas that are hard to reach areas where we have no doctors.” (P16)
**Aspect to be improved of the SkinNTDs app (n=7)**			
	Signs and symptoms section	4 (57)	“It is important to understand the sign and symptoms to don’t make wrong decisions. It may be not enough for nonexperts in Skin NTDs with the current information in the app. Hence, make wrong diagnosis.” (P1) “I think it is needed to add more images to correct identify correctly the signs/symptoms.” (P5)
	Customization aspect	1 (14)	“I think some holistic approach is needed to capture all skin NTDs.” (P6) “The app should provide full information about a condition and its management according to different levels of care.” (P9) “There is not an option to customize some preferences related to the App.” (P4)
	Issues to be solved	1 (14)	“There are still small issues to be solved, such as hyperlinks which do not work, specially related to the images. Moreover, there is not a direct connections with the developers to make them aware of it.” (P3)
	Lack of an expert panel	2 (29)	“I would like to have an option to upload photos, and ask a panel of experts to give their opinion in real-time.” (P2)
	Lack of African photos	2 (29)	“Most of the photos are referred to outside African countries.” (P9)
**How to improve customization aspect (n=4)**			
	Notifications	4 (100)	“In terms of notifications it may help to receive notifications when something is updated. I think this is the most significant part from customization, the notifications part.” (P1) “I would like to have the option to programm sounds and notifications.” (P10)
	Aesthetics	1 (25)	“I would like to have more options to customize some aspects, such as letter size or colors of the App.” (P4)
**How to improve interactivity aspect (n=4)**			
	Feedback	3 (75)	“Maybe it could improve by adding more feedback and reminders.” (P6) “The feedback and inputs should be reviewed on monthly basis.” (P10)
	Frequently asked question section	1 (25)	“Put a FAQ on the Skin NTDs APP could be beneficial.” (P7)
**Extra aspects (n=3)**			
	Dissemination strategy	3 (100)	“Liaise with the professional body, for example with professional association. This will be good way to disseminate.” (P13) “I think the first thing is creating awareness of the existence of the app through some kind of a seminar.” (P15) “It can also be introduced through the training institutions. You know can introduce it to them, they use it in training their students and then once they graduate, they go out well informed and then they can use it right away.” (P15) “The Ministry of Health takes it up and then it becomes as a standard of the way of managing patients with the skin diseases.” (P16)

^a^Not every participant provided sufficient elaboration to allow for identification of themes, so the frequency of individual themes does not add up to the total number of participants who participated in the semistructured interviews.

#### Best Features of the SkinNTDs App (n=10)

Most of the participants (n=8, 80%) considered that the best feature of the SkinNTDs app is how good it is for people without skin NTDs experience. They stated that the app gives them a wider understanding and helpful directions to obtain further knowledge about these conditions. In addition, 40% (n=4) of participants identified the app’s user-friendly interface and offline functionality as significant aspects. Participants also mentioned the use of simple language in the app (n=2, 20%) and highlighted its potential scope (n=2, 20%), particularly as a tool suitable for use in rural areas.

#### Points to Improve in the SkinNTDs App (n=7)

The signs and symptoms section was the most frequently mentioned area for improvement (n=4, 57%), especially with regard to understanding the definition of signs and symptoms, which could lead to misdiagnosis. Furthermore, 29% (n=2) of participants expressed concerns about the absence of an expert panel for addressing doubts and queries. In addition, they highlighted the limited availability of African photographs (2/7, 29%), which posed challenges in accurately identifying symptoms on dark skin. Finally, 1 (14%) participant suggested enhancing the app’s personalization aspect, while another pointed out unresolved issues that negatively impacted his experience with the SkinNTDs app.

#### How to Improve the Customization Domain of the SkinNTDs App (n=4)

In terms of customization, the worst-rated domain in the uMARS, all participants (n=4, 100%) unanimously identified the notifications as a key point to improve the customization of the app. Specifically, participants emphasized the need for features such as sound alerts or notifications to indicate app updates. In addition, 1 (25%) participant expressed the importance of having the option to customize the font size within the app.

#### How to Improve the Interactivity Domain of the SkinNTDs App (n=4)

The 2 requests from participants to improve the interactivity of the SkinNTDs app were to add feedback (n=3, 75%) and a frequently asked question section (n=1, 25%).

#### App Assessment Duration (n=11)

Of the 11 participants who responded to the question on whether 5 days for testing the app was sufficient time to apply the appropriate evaluation criteria, 8 (73%) participants stated that it was sufficient, while 3 (27%) participants indicated that they felt the testing period should be increased to between 10 and 15 days.

#### Medical Divide Feasibility (n=6)

When asked whether the SkinNTDs app could be used as a medical device in the future, 5 (83%) participants responded affirmatively. They cited the app’s ability to be easily used almost anywhere and its regular updates as the main reasons for their positive outlook. However, 1 (17%) participant believed that the current state of the app was not sufficiently developed to be considered a medical device. They suggested that further development and clinical validation would be necessary to consider that as a viable option.

#### Use of Other Health Apps (n=15)

The vast majority of participants (n=13, 86%) denied using any kind of health care app. In contrast, 2 (13%) participants admitted to using some form of health app. One participant reported using an app specifically designed for managing patients with tuberculosis; the other participant mentioned having used an app but could not recall its specific purpose.

#### Dissemination Strategy (n=3)

After addressing all the questions, 3 (100%) participants emphasized the importance of discussing the dissemination strategy for the SkinNTDs app. They unanimously agreed that official institutions, such as regional or national associations, universities, and the Ministry of Health, should be involved in the dissemination process. They stressed that involving these associations would help establish the app as a standard of care and ensure that future health care professionals are trained in using technology effectively in their daily work practices.

## Discussion

### Principal Findings

Skin NTDs are a subgroup of 13 NTDs that present primarily as skin lesions that can be detected through visual screening. Tackling these diseases depends on prompt diagnosis and treatment; hence, the resources used tend to be substantial. If they are not identified or treated, they could become chronic and irreversible, favoring a cycle of declining social, economic, and health conditions, which has a negative impact on the overall growth of LMICs [5,9].

Efforts to improve the diagnosis and management of skin NTDs are crucial, yet many existing strategies, despite showing effectiveness, are constrained by their face-to-face nature, focus on single conditions, and lack of sustainability and scalability [11,13-15]. Furthermore, rural areas still tend not to benefit from these interventions because they are often excluded [32]. Recognizing these challenges, the WHO highlights the significance of mHealth solutions, since it could offer a versatile and scalable approach to overcoming the limitations of traditional methods and more sustainable management of skin NTDs across diverse settings [19].

A study conducted by Krah and de Kruijf [25] revealed that in Africa, effective mHealth interventions are characterized by straightforward design and modest objectives, favoring simplicity over complicated interventions. This could explain why, according to the report, most mHealth interventions in the region are SMS text messaging based rather than app based and are primarily focused on disorders such as HIV and malaria as well as sexual and reproductive health. Along the same lines, a comprehensive evaluation of mHealth strategies for addressing skin NTDs indicated that most interventions are SMS text messaging based, and further effort is needed to homogenize therapies and eliminate methodological limitations [33]. The review by Carrion et al [33] identified only 2 interventions which were app based [16,17]: Skin App, developed by Netherlands Leprosy Relief and currently integrated into the SkinNTDs app evaluated in this study and Guaral App, developed by Universidad Icesi Grupo I2T. [2,11,13-15,25].

In order to increase the likelihood of success, it is vital to understand whether the proposed solutions are feasible in the real context and whether they are suitable for the end users [25]. It is therefore crucial to conduct a thorough evaluation of mHealth interventions in their different stages of development. Guaral App is designed to assist community health professionals in rural Colombia with the detection and referral of patients with cutaneous leishmaniasis, and as such, it is the only app found that is comparable to the SkinNTDs app. Furthermore, its usability has already been evaluated with positive findings, and sensitivity was found to be greater than 95%. However, because the researchers had recruited only 9 participants and did not use a validated measure to assess the usability, the results should be interpreted with caution [34].

The recently released eSkinHealth is another app that aims to enable onsite and remote diagnosis, monitoring, and clinical decision support for skin diseases, including NTDs, specifically designed for LMICs and darker skin types. Researchers plan to assess its usability with the System Usability Scale [32].

The SkinNTDs app developed by the WHO’s Department of Control of Neglected Tropical Diseases is currently in the early stages of its life cycle. Therefore, before moving on, it is crucial to assess at this stage whether it has been properly designed to fulfill its purpose, which is to be an educational and decision support tool to help frontline health care workers improve the management of NTDs in their daily practice. According to WHO guidelines, it is at the stage when its feasibility and usability should be evaluated [24].

The uMARS tool was chosen to assess version 3 of the SkinNTDs app, as it has already been widely used to evaluate other health apps, and it has proven its simplicity and reliability [26]. In addition, semistructured interviews were undertaken to complete the study from all perspectives.

Before discussing the results in more detail, it is important to note that the sample includes both frontline health care workers and non–frontline health care workers. The link to participate in the study also reached other health professionals who were not classified as frontline health care workers. Although it was initially thought that the sample should be restricted to actual end users, responses of non–frontline health care workers were also recorded, allowing for a more detailed analysis. Since there were no differences between these 2 groups in any of the variables, it was decided to include them in the study and ensure greater consistency in the findings.

The results of this study show that the SkinNTDs app has a high level of quality as measured by the uMARS questionnaire, obtaining an app quality mean score of 4 (SD 0.47) of 5, a subjective quality score of 3.83 (SD 0.61) of 5, and a perceived impact of 4.5 (SD 0.56) of 5. These results seem to indicate that the app meets the users’ needs.

Perhaps the most important result obtained in this study is that there is neither difference nor correlation between any variables with the app quality mean score. This may suggest the potential feasibility of the app being widely distributed to countries affected by skin NTDs without requiring any customization other than making new languages available. Some articles have highlighted the importance of the effective use of innovative technologies in health care and the development of sustainable strategies [35]. The SkinNTDs app is obviously an example of a case that appears to have great potential for dissemination without a significant financial investment. Another important finding is that 84.1% (50/60) of participants rated the app with a 4 or 5 for ease of use, although most (47/50, 94%) of them did not receive any training before using it. This fits well for the design of any future dissemination campaign, as the app could be offered without prior training, saving money on implementation. The worst-rated domains could be enhanced by the app developers with some changes. In particular, improving the customization subdomain (the worst rated) could help the app to adapt to the priorities of each user.

Participants also expressed interest in adding other capabilities to the app, such as patient records, surveillance information for skin NTDs, or a desktop version. Something important to bear in mind when building these potential new features is that 30.7% (16/60) of participants reported having a poor internet connection.

Regarding the second part of the study, in which the semistructured interviews and focus group were conducted, 56% (34/60) of participants showed interest in participating and were contacted by email. Despite sending numerous reminders to all of them, only 17 people were finally interviewed, thanks to the efforts of 2 key actors and researchers from this study (EK and PM), without whom the second part of this study could not have been conducted. However, the web-based interviews were challenging due to unreliable internet connections, which hindered the smooth flow of conversation. As a result, not all participants were able to provide responses to all the questions. These facts will be further discussed in the following Limitations section. Nevertheless, most of the participants’ answers corroborated the results obtained in the first part of the study with the uMARS questionnaire.

Once again, the best features of the app were considered to be the target group (frontline health care workers), the user-friendly interface, the offline functionality, the use of simple language, and its potential scope. The target user group is especially important to mention, since the goal of this app is to train workers who deal with these conditions but do not have in-depth knowledge of them.

In contrast, the participants also identified some points that should be reviewed in order to improve the whole experience with the app. The sign and symptoms section was where the participants found the most issues that could be improved, mainly because understanding them is vital for a correct diagnosis. This is directly related to the group of people who are expected to use the app and lack the necessary knowledge or experience to correctly identify the signs and symptoms if these are not clearly explained. Thus, this is a major point to consider. Another possible weakness in the signs and symptoms section is the use of photos that are not specific to the region where the app is used. In this case, participants mentioned the lack of African photos, which posed challenges in accurately identifying symptoms on dark skin.

Participants reached a consensus regarding the potential improvements to the 2 weakest domains, customization and interactivity. They suggested that incorporating notifications, sounds, and a frequently asked question section could address many of their concerns. In addition, they emphasized the importance of seeking feedback from experts. However, it is crucial to carefully consider this suggestion, as implementing web-based functionalities may compromise one of the key advantages of this app, as highlighted by the participants: the offline functionality, which enables effective dissemination in areas with limited connectivity.

Regarding the classification of the app as a medical device, the consensus among participants was that achieving this status could be feasible with future updates, further development, and rigorous validation. This would mean that the SkinNTDs App could expand its utility beyond merely training frontline health care workers, serving additionally as a diagnostic tool. However, this step could be very relevant, considering the positive impact that some projects incorporating artificial intelligence tools in LMIC have had. However, there are still numerous challenges and limitations that need to be taken into account beforehand, such as reliability issues, impacts on workflows, ease of use, and the importance of local context in the effectiveness of artificial intelligence tools [36].

Finally, participants deemed it important to discuss the most effective dissemination strategy for the app. Given that the WHO views this app as a valuable approach to addressing skin NTDs in LMICs, it is worth noting that only 2 participants in the study reported using other health apps in their work. Consensus was reached among all participants that the most favorable approach would be to collaborate with official institutions for dissemination purposes. However, while a marketing strategy could also be considered, studies affirm that health care marketing requires its own approach due to the unique features of the industry [37].

Considering that the app is still in development and has not yet been widely used in countries where skin NTDs are common, this study presents the opportunity to include end user feedback in future editions of the app. Furthermore, the impact of any subsequent implementation campaigns is likely to be influenced by this action.

### Limitations

There were various limitations associated with this study that should be considered.

The first limitation was the decision to perform this study in Ghana and Kenya, based on 3 factors: (1) when the study was carried out, the SkinNTDs app could only be downloaded in English; hence, the choices were limited to countries where English is an official language; (2) t2 countries have a long history of web-based disease tracking and at least 8 skin NTDs are endemic in each; and (3) to ensure adequate sampling, countries were chosen based on the accessibility of key officials and program managers and their ability to contact enough respondents.

Regarding the methodology used, snowball sampling is a nonprobabilistic and nonrandomized method; thus, the sample may not be representative. However, because the study was conducted entirely on the web and the participants and authors were based in different places, this was the only reasonable way we could connect with frontline health care workers —a highly specialized group that can be challenging to reach in LMICs if not contacted remotely. The truth, however, revealed that this recruitment procedure was still challenging and would not have been accomplished without the direct assistance of 2 key actors from these 2 countries.

In addition, it is also important to point out some limitations of uMARS. First, this tool has only been validated in young people using 2 specific apps related to other health areas [26]. However, its high reliability and the inclusion of both objective and subjective domains make it the only validated and simple questionnaire available for users to assess health apps.

Another requirement is that the frontline health care workers had to use the app for at least 5 days as opposed to the 10 minutes required by the original uMARS publication. Since there was no direct control over this requirement being met, this aspect could not be guaranteed. We asked participants during the semistructured interviews whether 5 days was enough time to test the app before answering the uMARS, and most (8/11, 73%) of them agreed that it was.

Several complications arose during the second semistructured part of the study, mainly due to not being able to carry out the interviews onsite because of COVID-19–related lockdowns and travel restrictions. The first issue was that no matter how many reminders were sent to the participants who agreed to be interviewed, only 17 finally participated. In fact, all those who participated in this part of the study were contacted directly by these 2 key agents. Moreover, during the web-based semistructured interviews, other complications appeared, such as participants constantly dropping in and out of the video call because they lost their internet connection, making it very difficult to create a warm environment for them to be more engaged. Similar difficulties arose in the focus group, and the expected interactions between the participants did not occur. As a result, many of them did not discuss their answers, leading to the exclusion of those responses from the study. It is important to acknowledge that this directly impacted the quality of the app’s qualitative evaluation.

Finally, it is important to highlight that this study did not intend to assess the clinical effectiveness of the SkinNTDs app. This would require a randomized clinical trial and exceed the scope of this study. Although this study evaluated version 3, there are still issues to address, including whether information in the app is updated in response to the most recent research.

### Conclusions

The SkinNTDs app represents a significant step forward in the use of mHealth technologies for the management of skin NTDs in LMICs. This study’s findings that highlight the app’s good usability and usefulness suggest its potential to be used as a training and support tool for frontline health care workers, without necessitating extensive customization. However, future development efforts must address identified weaknesses and consider user feedback to enhance the app’s effectiveness and user satisfaction. By doing so, the SkinNTDs app can play a pivotal role in the global effort to combat skin NTDs and reduce their burden on affected communities.

In addition, its potential as a diagnostic tool merits exploration, and further research must evaluate this tool’s clinical efficacy and determine the optimum method for achieving global diffusion.

## Appendix

Multimedia Appendix 1 Deviations from the study protocol.

Multimedia Appendix 2 User version of the Mobile Application Rating Scale raw data derived from the cross-sectional study.

Multimedia Appendix 3 Data derived from the semistructured interviews and focus groups.

## Abbreviations

LMICs: low- and middle-income countries
mHealth: mobile health
NTD: neglected tropical disease
uMARS: user version of the Mobile Application Rating Scale
WHO: World Health Organization

## Data Availability

The data sets generated during this study are available to be downloaded in the multimedia appendix. Specifically, Multimedia Appendix 2 contains data derived from the user version of the Mobile Application Rating Scale (uMARS), while Multimedia Appendix 3 features key responses from participants gathered through semistructured interviews or focus groups. All data considered confidential have been excluded from these resources.
