# Supplementary material for: Evaluating the World Health Organization’s SkinNTDs App as a Training Tool for Skin Neglected Tropical Diseases in Ghana and Kenya: Cross-Sectional Study
Source: J Med Internet Res. 2024 Apr 30;26:e51628. doi: 10.2196/51628 (PMC11094592; doi:10.2196/51628)
Supplement: Multimedia Appendix 1 [file jmir_v26i1e51628_app1.docx]

Multimedia Appendix 1. Deviations from the study protocol

| **Planned approach described in the protocol** | **Type, description and justification of the deviation from the protocol** |
| --- | --- |
| We planned to include only FHW and exclude non-FHW. | Type of deviation: change  We decided to include non-FHW because there was no statistical difference between the app quality mean score vs the type of health worker |
| We planned to do a cluster analysis to identify similar groups based on the observed values of several variables | Type of deviation: change  We did not perform a cluster analysis because we found no associations between variables. |
| We planned to do 7 core questions during the semi-structured interviews. | Type of deviation: change  First of all, after obtaining the results from the first part of the study, we believed it was necessary to know why the customization subdomain was the worst rate. That is why we added a question about it.  Secondly, while the difficulties arose during the semi-structured interviews regarding the connection problems and the difficult interactivity or understanding with some of the participants, it was necessary to change some of the questions to keep it simple. |
| We planned to do semi-structured interviews | Type of deviation: change  Even though we achieved to conduct 10 semi-structured interviews, due to recruitment difficulties it was necessary to conduct a focus group in Kenya to increase the sample size and achieve the saturation of the information. |
