# Supplementary material for: Evaluating the World Health Organization’s SkinNTDs App as a Training Tool for Skin Neglected Tropical Diseases in Ghana and Kenya: Cross-Sectional Study
Source: J Med Internet Res. 2024 Apr 30;26:e51628. doi: 10.2196/51628 (PMC11094592; doi:10.2196/51628)
Supplement: Multimedia Appendix 3 [file jmir_v26i1e51628_app3.docx]

**Multimedia Appendix 3 features key responses from participants gathered through semi-structured interviews or focus groups.**

**What do you think are the best points of the App? Why?**

- P01. “The App gives you a wide understanding about SkinNTDs without being an expert in this topic”
- P02. “Is nice to have this App always available with you, even when you don’t have access to internet”
- P03. “What I really appreciate from the App is that is really easy to navigate”
- P03. “The App helps people without knowledge in SkinNTDs and leads them to the right directions to make diagnosis”
- P03. “Is great to have all the information in an App”
- P04. “What I really appreciate from the App is that is really easy to navigate”
- P04. “The App helps people without knowledge in SkinNTDs and leads them to the right directions to make diagnosis”
- P04. “Is great to have all the information in an App”
- P11. “The app adds value, especially during diagnosis of tropical and forgotten diseases. For example, sometime back before the App, it was very difficult to make a diagnosis […]. But now, because of the App, it's easy to make that diagnosis. so it's beneficial.
- P12. “It doesn't need any additional resources because people have their phones best points”
- P13. “From my experience using the app I found it very easy to use and very fast. It has a lot of information about some diseases which is also very interesting to use.
- P14. “The App is nice, because I can see it has given us information, so it is easy to diagnose best points”
- P14. “So it is good because, many people can be able to get the app, they can be able to use the app, and so that is nice”
- P15. “Yes, I think by use of this App it has brought back the sense of thinking about the neglected tropical diseases. Personally I had just even forgot about some of these diseases and now with the introduction of the App, it has taken me back and I'm all now looking at some of these diseases are there only that we have not been having sufficient time and also being about taking for example the issue of the Leishmaniasis which is not very common here but still it has given me an opportunity to refresh my mind on how this Leishmaniasis, the clinical features and also how one can be able to diagnose it. best points”
- P16. “I think the app can be used especially in those areas, remote areas that are hard to reach areas where we have no doctors. So with the kind of information that is there I think its future it can easily be based on someone else”
- P17. “I believe the app is beneficial because you can almost make a spot diagnosis now, depending on the type of what you see, because there are some which are not very easy to differentiate, but now from the pictures it is kind of clearer”

**What aspects of the SkinNTDs application could be improved? Why?**

- P01: “It is important to understand the sign and symptoms to don’t make wrong decisions. It may be not enough for non-experts in Skin NTDs with the current information in the app. Hence, make wrong diagnosis”
- P02: “I would like to have an option to upload photos, and ask a panel of experts to give their opinion in real-time””
- P03: “There are still small issues to be solved, such as hyperlinks which do not work, specially related to the images.Moreover, there is not a direct connections with the developers to make them aware of it”
- P04: “There is not an option to customize some preferences related to the App”
- P05: “I think it is needed to add more images to correct identify correctly the signs/symptoms”
- P06: “I think some holistic approach is needed to capture all skin NTDs”
- P08. “There are not enough photos of African people”
- P09: “The app should provide full information about a condition and its management according to different levels of care”
- P09: “Most of the photos are referred to outside African countries”
- P12. “ It could be nice if you can take a picture of a skin condition and upload for somebody to help you review it for you”

**How could we improve the customization aspect of the App?**

- P01: “In terms of notifications it may help to receive notifications when something is updated. I think this is the most significant part from customization, the notifications part”
- P04: “I would like to have more options to customize some aspects, such as letter size or colors of the App”
- P06. “In my opinion content and notifications should be improved”
- P08. “It is needed to have notifications”
- P10: “I would like to have the option to programm sounds and notifications”

**How could we improve the interactivity aspect of the App?**

- P06: “Maybe it could improve by adding more feedback and reminders”
- P07:” Put a FAQ on the Skin NTDs APP could be beneficial”
- P10: “The feedback and inputs should be reviewed on monthly basis”

**Would you like to add something else, or talk about another aspect that you feel is relevant to the app?**

- P13: “Liaise with the professional body, for example with professional association. This will be good way to disseminate”
- P15: “I think the first thing is creating awareness of the existence of the app through some CMEs or some kind of a seminar such that you have the first team to be able to understand what the app is all about”
- P15: “I think they can also introduce it through the training institutions. For instance, looking at KMTC you know can introduce to them they use it in training their students and then once they graduate and once it is would have been made a policy as she said this pool of healthcare providers will go out well informed and then they can use it outright”
- P16: “How I think if it's made somehow becomes like a policy or something like that such that the Ministry of Health takes it up and then it becomes as a standard of the way of managing patients with the skin diseases. So first of all, it has to come up like it is a policy that, this is an app that should be used in the healthcare facilities so that once it's like that, then the people who are in those rural areas will be able to use the app.
